# Extraction and Identification of Phlorotannins from the Brown Alga, *Sargassum fusiforme* (Harvey) Setchell

**DOI:** 10.3390/md15020049

**Published:** 2017-02-21

**Authors:** Yajing Li, Xiaoting Fu, Delin Duan, Xiaoyong Liu, Jiachao Xu, Xin Gao

**Affiliations:** 1 College of Food Science & Engineering, Ocean University of China, Qingdao 266000, China; Sakura_yj@163.com (Y.L.); xujia@ouc.edu.cn (J.X.); xingao@ouc.edu.cn (X.G.); 2 Key Laboratory of Experimental Marine Biology, Institute of Oceanology, Chinese Academy of Sciences, Qingdao 266071, China; dlduan@qdio.ac.cn; 3 State Key Lab of Seaweed Bioactive Substances, Qingdao 266000, China; 4 Shandong Haizhibao Ocean Science and Technology Co., Ltd., Qingdao 266000, China; xyliu@chengshan.com

**Keywords:** *Sargassum fusiforme*, brown alga, phlorotannins, polyphenol, extraction method, identification, UHPLC-DAD-ESI-MS

## Abstract

Phlorotannins are a group of complex polymers of phloroglucinol (1,3,5-trihydroxybenzene), which are unique compounds from marine brown algae. In our present study, a procedure for extraction and enrichment of phlorotannins from *S. fusiforme* with highly antioxidant potentials was established. After comparison of different extraction methods, the optimal extraction conditions were established as follows. The freeze-dried seaweed powder was extracted with 30% ethanol-water solvent with a solid/liquid ratio of 1:5 at temperature of 25 °C for 30 min. After extraction, the phlorotannins were fractioned by different solvents, among which the ethyl acetate fraction exhibited both the highest total phlorotannin content (88.48 ± 0.30 mg PGE/100 mg extract) and the highest antioxidant activities. The extracts obtained from these locations were further purified and characterized using a modified UHPLC-QQQ-MS method. Compounds with 42 different molecular weights were detected and tentatively identified, among which the fuhalol-type phlorotannins were the dominant compounds, followed by phlorethols and fucophlorethols with diverse degree of polymerization. Eckol-type phlorotannins including some newly discovered carmalol derivatives were detected in *Sargassum* species for the first time. Our study not only described the complex phlorotannins composition in *S. fusiforme*, but also highlighted the challenges involved in structural elucidation of these compounds.

## 1. Introduction

Phlorotannins are a class of polyphenol compounds produced by brown seaweed as secondary metabolites and biosynthesized via the acetate malonate pathway [1,2,3]. They are present in the algae in free form or forming complexes with different components of the cell walls, such as alginic acid [2,4]. Phlorotannins are essential to the physiological integrity of alga and involved in a number of important secondary roles such as chemical defense, protection against oxidative damage that occurs in response to changes in nutrient availability and UV radiation, interactions with other organisms or the abiotic environment, as well as being integral components of cell wall [5,6]. These compounds have attracted considerable research interest for their broad health benefits and potential uses in a range of therapeutics [4,7].

Phlorotannins are basically composed of phloroglucinol (1,3,5-trihydroxybenzene) units with different degrees of polymerization (DP) and a group of heterogeneous polymeric compounds. In general, based on the means of linkage, phlorotannins are classified into four subclasses, i.e., phlorotannins with ether linkages (fuhalols and phlorethols), those with phenyl linkages (fucols), those with both ether and phenyl linkages (fucophlorethols), and those with a dibenzodioxin linkage (eckols) [8,9]. Fuhalols differ from phlorethols by the presence of additional hydroxyl groups. The phlorotannins composition in algae is always of high complexity [4,10]. As reported by Montero et al. [4], 53 species of phlorotannins belonging to fuhalol and phlorethols were detected in the *Sargassum muticum* collected in Norway. Furthermore, even at the same molecular weight, it is possible to have various positions at which a bond can occur between monomers, leading to many structural isomers in addition to conformational isomers [10]. As reported by Heffernan et al. [10], a high number of isomers were detected for individual molecular ions in the species *Fucus serratus*, with 178 isomers in total being detected from DP of 6 to 14. Although their presence in brown algae is widely accepted, relatively limited characterization of such complex polymeric structures has been carried out [4].

*Sargassum fusiforme* (Harvey) Setchell, also known before as *Hizikia fusiformis* [11] is a famous brown seaweed, which has been cultivated in China, Korea, and Japan and consumed as food for thousands of years. In Japan, *S. fusiforme* is called “longevity greens”, which is imported from China in a large quantity every year. It is used as a traditional Chinese herbal medicine which has been documented in Compendium of Materia Medica. The bioactivities of crude extracts from *S. fusiforme* were studied, and the results indicated that *S. fusiforme* possessed remarkably high antioxidant [12], anti-inflammatory [13,14], anti-allergic [14], antimicrobial [15], anti-diabetic [13], and HIV-1 inhibitor activites [16]. However, few studies were carried out to estimate the bioactivities of phlorotannins extracts from this alga. To our knowledge, there is no report on isolation and structural characterization of phlorotannins from *S. fusiforme.*

In our preliminary study, it was found that the phlorotannins constitute up to 6% of the dry weight of *S. fusiforme* collected from Dongtou, Zhejiang province. The aim of the current study was to establish a procedure for isolation and enrichment of phlorotannins from *S. fusiforme*, determine their antioxidant activities, and investigate the phlorotannin composition by using ultra-high performance liquid chromatography (UHPLC) with triple quadrupole tandem mass spectrometry (UHPLC-QQQ-MS). The UHPLC-QQQ-MS analysis of the extracts determined the complex phlorotannins composition of fuhalols, fucophlorethols, phlorethols, and eckols. There are several reports about similar research work for macroalgae, such as *F. serratus* [1], *Macrocystis pyrifera* [2], and *S. muticum* [4], but this is the first time to the isolation and characterization of phlorotannins from *S. fusiforme* has been intensively studied. It is also the first time that eckol-type phlorotannins, including some new carmalol derivatives, have been discovered in *Sargassum* species. The information obtained in this study will help to understand the relationship of structure and activities of phlorotannins from *S. fusiforme* and promote its potential application in functional food and the pharmaceutical industry.

## 2. Results and Discussion

### 2.1. Determination of Extraction Parameters

#### 2.1.1. Effect of Solvent Concentration

Ethanol, methanol, and acetone are always selected for phlorotannin extraction. On the basis of our pretests (data not shown) and other studies [17,18,19], ethanol showed considerable ability to extract polyphenols from both seaweeds and terrestrial plants. The use of ethanol would obviously be preferred over acetone and methanol for the extraction of both food-grade and pharmaceutical-grade natural antioxidants [1]. It was also reported that a binary solvent system containing hydro-organic solvents was superior to a mono-component solvent system (pure water or ethanol) in the extraction of phenolic compounds for the synergistic effect [20,21]. The mixture of water and ethanol as extraction solvent was therefore chosen for further studies.

**Figure 1:**
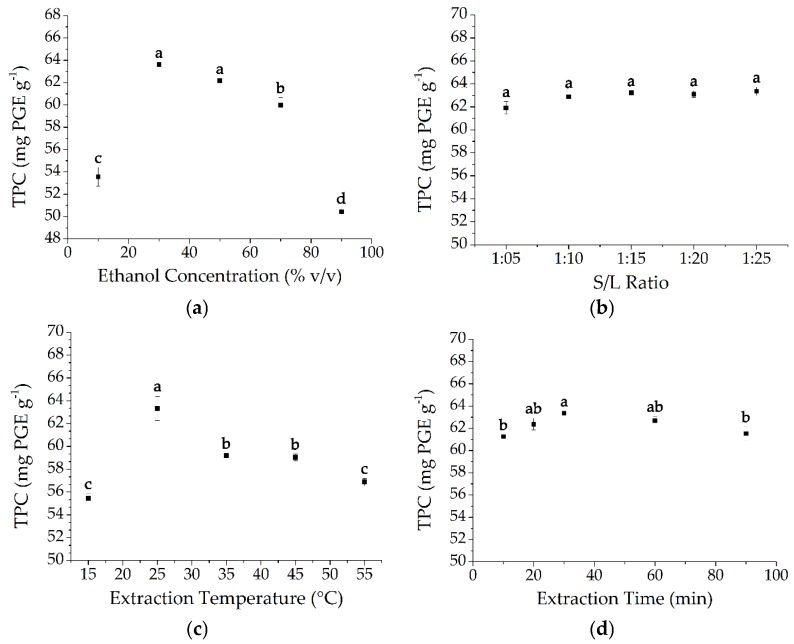
Evaluation of the effects of (**a**) ethanol concentration; (**b**) solid/liquid ratio; (**c**) extraction temperature; and (**d**) extraction time on the TPC in *S. fusiforme*. The letters on the scatters indicate statistically different groups (*p* < 0.05).

The influence of solvent concentration on the total phlorotannin content (TPC) was shown in Figure 1a. The results revealed that the yield of phlorotannins increased significantly from 10% ethanol (53.57 ± 0.85 mg PGE·g^−1^, PGE means phloroglucinol equivalents) to 30% ethanol (63.61 ± 0.16 mg PGE·g^−1^), but then decreased with the further increasing of the ethanol proportion. The value of TPC was maximized at 30% ethanol (63.61 ± 0.16 mg PGE·g^−1^) and followed by 50% ethanol (62.17 ± 0.06 mg PGE·g^−1^), but no significant difference was observed between them (*p* = 0.168). Ethanol is a relatively low-polar solvent while water is a strong polar solvent, so the polarity of the extraction solvent will decrease continuously with the addition of ethanol to water [17]. The result that maximum TPC was obtained at 30% ethanol indicated that the phlorotannin molecules in *S. fusiforme* were polar. Finally, 30% ethanol was selected as the extraction solvent for its lower cost in industrial application compared with 50% ethanol.

#### 2.1.2. Effect of Solid/Liquid Ratio

The extractions carried out at different solid/liquid ratios were conducted and the results were shown in Figure 1b. The results indicated that the yield of TPC slightly increased from 1:5 ratio (61.91 ± 0.54 mg PGE·g^−1^) to the highest value at 1:25 ratio (63.36 ± 0.35 mg PGE·g^−1^). However, there was no significant difference between the values. The results were in accordance with the previous findings that a higher solid/liquid ratio lead to a higher yield of polyphenols [19,20]. It should be noted that a higher liquid ratio generated not only an increase in the consumption of ethanol and water thus the cost of extraction, but also a longer concentration process, which might improve the oxidation of phlorotannins. Therefore, a solid/liquid ratio of 1:5 was selected in this study.

#### 2.1.3. Effect of Extraction Temperature

The yields of TPC at different temperature were shown in Figure 1c. The extraction efficiency regarding TPC increased significantly from 15 to 25 °C, but then decreased significantly as the extraction temperature increased from 25 to 55 °C. In our knowledge, the increasing of temperature could help the release and dissolution of polyphenol compound, but high temperature could also promote degradation such as the oxidation of these compounds and increase the extraction of protein and polysaccharides from the cell wall [17,18]. In many studies of some other seaweeds and terrestrial plants including *Sargassum serratum* [19], *M. pyrifera* [2], and *Saccharum officinarum* [22], the optimum extraction temperature obtained was about 50 °C, and this was the reason why we chose this temperature as the initial extraction condition (see, Section 3.3.1). While in our study, it seemed that polyphenols from *S. fusiforme* were more suitable for extraction at room temperature rather than relatively higher temperature. The thermolability of phlorotannins from *S. fusiforme* might due to its excellent antioxidant activity and extremely complex composition (see, Section 2.2 and Section 2.3). Finally, the extracting temperature of 25 °C was selected.

#### 2.1.4. Effect of Extraction Time

The effect of extraction time on the TPC was shown in Figure 1d. The extraction efficiency was significantly improved as the duration of the extraction increased, and reached a maximum at 30 min (63.35 ± 0.19 mg PGE·g^−1^) and then decreased thereafter. The probable reason might be that the longer the extraction time was, the more severe the oxidation happened to phlorotannins was. The phlorotannins in *S. fusiforme* were easier to extract compared to those from other seaweeds like *S. serratum* [19] and *M. pyrifera* [2], whose best extraction time was no less than 4 h. In our study, we chose 30 min when TPC reached the maximum level for further research.

Thus, the optimal extraction conditions were finally established to be 30% ethanol-water mixture as solvent, a solid/liquid ratio of 1:5, temperature of 25 °C, and extraction time of 30 min.

### 2.2. Total Phlorotannin Content (TPC) and Antioxidative Activity of 30% Ethanol Extract and Its Solvent Fractions

**Figure 2:**
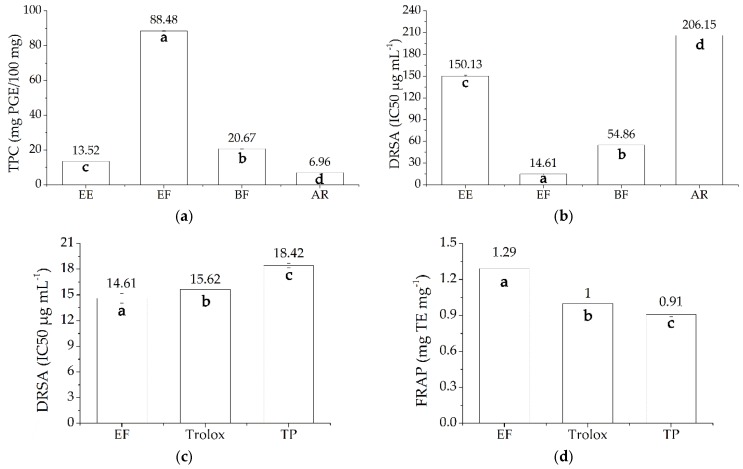
The (**a**) TPC and (**b**) DRSA of different solvent fractions of *S. fusiforme* and the comparison of (**c**) DRSA and (**d**) FRAP of ethyl acetate fraction with trolox and commercially available tea polyphenols with a purity of 90%. EE, ethanol extracts; EF, ethyl acetate fraction; BF, 1-butanol fractions; AR, the aqueous residue; Trolox, a synthetic antioxidant analogous to vitamin E; TP, the commercially available tea polyphenols with a purity of 90%. The letters on the bar indicate statistically different groups (*p* < 0.05).

The 30% ethanol extract was subsequently partitioned into three fractions—ethyl acetate and 1-butanol soluble fractions and the final aqueous residue—by liquid-liquid extraction. The TPC and DPPH radical scavenging activity (DRSA) of crude ethanol extracts and each fraction were shown in Figure 2a,b, respectively.

There were significant differences in TPC among the 30% ethanol extract and its subsequent fractions (Figure 2a). The results indicated that after liquid-liquid extraction, the TPC in ethyl acetate fraction and 1-butanol fraction were higher than that of the initial ethanol extract, while the TPC in aqueous residue was lower than that of the initial ethanol extract. The highest level of TPC was found in the ethyl acetate fraction with a value of 88.48 ± 0.30 mg PGE/100 mg, which was dramatically higher than that in 1-butanol fraction and aqueous residue whose TPC values was at 20.67 ± 0.08 and 6.96 ± 0.02 mg PGE/100 mg, respectively. Ethyl acetate has been widely used to selectively extract polyphenolic compounds from various seaweeds [8,23,24,25,26,27,28]. TPC in the ethyl acetate fraction of *S. fusiforme* was similar to that of *Fucus vesiculosus* (88.3 ± 2.2 mg PGE/100 mg) reported by Wang et al. [1], but dramatically higher than that of many other species including *Eisenia bicyclis* [29,30,31], *Ecklonia maxima* [32], and *S. muticum* [4]. The present study showed that it was also effective in enriching phlorotannins from the crude extract of *S. fusiforme*. As shown in Figure 2b, a similar result was observed in the DRSA test. The ethyl acetate soluble fraction also exhibited the highest scavenging activity (IC50 14.61 ± 0.56 μg·mL^−1^), followed by the 1-butanol fraction (IC50 54.86 ± 0.09 μg·mL^−1^), while the crude extract (IC50 150.13 ± 1.20 μg·mL^−1^) and aqueous residue fraction (IC50 206.15 ± 0.08 μg·mL^−1^) were much less effective. Pearson correlation analysis showed the significant positive correlation between TPC and DRSA of the 30% ethanol extract and its solvent fractions (*r* = 0.997, *N* = 4, *p* = 0.003), which indicated that phlorotannins are the major antioxidant components present in *S. fusiforme*.

The antioxidant activities including DRSA and FRAP of ethyl acetate fraction, Trolox (a synthetic antioxidant analogous to vitamin E), and a commercially available tea polyphenols with a purity of 90% were analyzed and the results were shown in Figure 2c,d, respectively. The results indicated that the antioxidant activity of the ethyl acetate fractions of *S. fusiforme* were significantly higher than those of commercial antioxidants. The DRSAs of crude extraction and ethyl acetate fractions from *S. fusiforme* in our study were both higher than that reported in previous studies for *S. fusiforme*, in which the different extraction methods were used. The results indicated that our extraction method was more efficient [33,34,35,36]. In addition, the DRSA of ethyl acetate fractions of *S. fusiforme* in our study (IC50 14.61 ± 0.56 μg·mL^−1^) was higher than those of some *Sargassaceae* species including *Halidrys siliquosa* (IC50 0.020 ± 3.54 × 10^−4^ mg·mL^−1^) [28] and *Cystoseira nodicaulis* (IC50 14.00 ± 0.04 μg·mL^−1^). When compared with other brown seaweeds, it was lower than that of *F. vesiculosus* (IC50 4.00 ± 0.01 μg·mL^−1^), similar to that of *Himanthalia elongate* (IC50 14.00 ± 0.04 μg·mL^−1^), and stronger than that of *F. serratus* (IC50 14.00 ± 0.04 μg·mL^−1^). The FRAP of ethyl acetate fractions from *S. fusiforme* was higher than many seaweeds, including *C. nodicaulis*, *F. vesiculosus*, *H. elongate*, and *F. serratus* in the study from Heffernan et al. [10] and seaweeds including *Ascophyllum nodosum*, *F. spiralis*, and *Pelvetia canaliculata* in the study from Tierney et al. [5]. Thus, the phlorotannins from the ethyl acetate fractions of *S. fusiforme* could be employed in the pharmaceutical industry as potent marine antioxidants.

### 2.3. UHPLC-QQQ-MS and MS/MS Profiling of the Phlorotannin-Enriched Fraction

**Figure 3:**
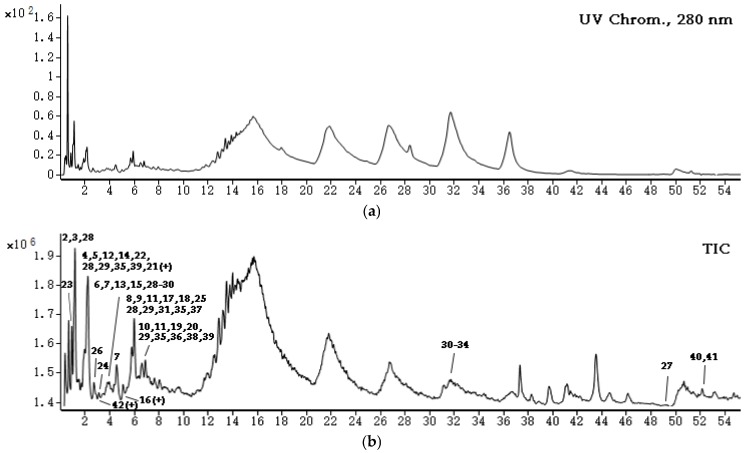
The (**a**) UV chromatograms recorded at 280 nm and (**b**) TIC of the ethyl acetate fraction. Peaks marked with numbers were tentatively identified using UHPLC-QQQ-MS, which were shown in Table 1. Numbers with (+) were detected in positive mode; the others were detected in negative mode.

The structure of matter often determines its activity and thus the application. Therefore, it is of great significance to characterize the composition of phlorotannins in *S. fusiforme* for better understanding of its activity and further development of its application. The ethyl acetate fraction exhibited both the highest TPC and the highest antioxidant activities, thus it was further analyzed using a UHPLC-DAD-ESI-MS. Figure 3 showed the UV chromatograms recorded at 280 nm and the total ion chromatogram (TIC) of the ethyl acetate fraction.

The complexity of phlorotannins—which have been reported to include fucols, phlorethols, fucophlorethols, fuhalols, eckols, and so on—can be attributed to not only the type of structural linkages which result from the polymerization process, but also the modification site and number of additional hydroxyl groups [37]. The identification of phlorotannins is difficult due to their various chemical structures and complex chemical composition in the algae [4,10]. The exact structure of phlorotannin isolated is always elucidated by a combination of both MS and NMR in many previous studies [38,39,40]. In our present study, the preliminary MS analysis was conducted and the composition of phlorotannins in *S. fusiforme* was determined. 42 different molecular weights with a DP of 2 to 12 could be detected and analyzed in *S. fusiforme* extracts, and their MS and MS/MS data were listed in Table 1. Fuhalol-type phlorotannins (No. 1–20) were found to be the most frequently elucidated components, and other phlorotannins classes—including phlorethols, fucophlorethols (No. 21–27), and eckols (No. 28–42)—with diverse degree of polymerization were also present.

The term “fuhalol” refers to compounds comprising ether-linked phloroglucinol units which contain an extra hydroxyl group on one unit, making that unit vicinal trihydroxylated [41]. The ether bonds are oriented para and ortho, and in most cases, the vicinally trihydroxylated ring bears ortho-phenyl ethers [41]. The number of additional hydroxyl groups in fuhalols is related to the degree of the polymerization and is equal to the biggest integer of the half of its DP [42,43]. For example, the octafuhalol and the nonafuhalol both have four hydroxyl groups. In this study, numbers 1 to 11 (Table 1) referred to bifuhalol to dodecylfuhalol, respectively. Among them, the most predominant peak was the bifuhalol, followed by the trifuhalol and tetrafuhalol. Some ether-linked phloroglucinol polymers that have fewer additional hydroxyl groups when compared to fuhalols with same degree of polymerization—such as deshydroxyfuhalols [43], hydroxyphlorethols [38,44], hydroxyfucophlorethol [45], and phlorethofuhalols [46]—also belong to fuhalol-type phlorotannins, and their molecular weights are at least one oxygen atom mass less than the molecular weights of corresponding fuhalols. In this study, numbers 12 to 20 (Table 1) with fewer additional hydroxyl groups compared to the corresponding fuhalols, might have one or more of the above structures and also some unknown structures. Fuhalol-type phlorotannins were mainly found in some *Sargassum* species like *S. spinuligerum*, *S. muticum*, and *S. ringgoldianum*, some *Sargassaceae* species such as *H. siliquosa*, *Cystoseira baccata*, and *Bifurcaria bifurcata*, and also some other brown seaweeds like *Chorda filum* and *Landsburgia quercifolia* et al. [25,28,47,48,49].

Fucols, phlorethols, and fucophlorethols are characterized by being formed from phloroglucinol units linked through phenyl bonds, ether bonds, and both ether and phenyl bonds, respectively [8,9]. Most of them do not have any additional hydroxyl groups, although there are some exceptions [24,25]. Thus fucols, phlorethols, and fucophlorethols with the same degree of polymerization will have same molecular weight and cannot be distinguished only by one-dimensional MS. In our present study, based on the MS/MS data (Table 1) as well as literature data [1,4], numbers 21 to 23 were categorized as phlorethols or fucophloroethols, for showing fragmentation patterns characteristic of ether bonds, with losses of phloroglucinol and oxygen atom (−125 amu and −16 amu) and two phloroglucinol and oxygen atoms (−265 amu). However, the contents of numbers 24 to 27 were too low to detect their fragmentation patterns, so they might belong to one or more of the above three classes of phlorotannins. The identification of these compounds still need further isolation and NMR analysis.

When there is at least one three-ring moiety with a dibenzodioxin elements substituted by a phenoxyl group at C-4 in the structure, the group is named eckols [10]. Up to now, eckol-type phlorotannins have been found mainly in the *Ecklonia*, *Eisenia, Ishige okamurae*, and *Carpophyllum maschalocarpum* [25,46,50]. In this class, eckols (M*_R_*: 372), dioxinodehydroeckol (M*_R_*: 370), tetramers phloroeckol (M*_R_*: 496), hexamers dieckol (M*_R_*: 742), and many other eckol derivatives such as fucofuroeckols, phlorofucofuroeckols, hydroxyeckols have been successfully isolated and elucidated [25,46,50]. Besides, two phlorethol-carmalol derivatives, diphlorethohydroxycarmalol (M*_R_*: 512) and triphlorethohydroxycarmalol (M*_R_*: 636), were isolated and elucidated in *I. okamurae* and *C. maschalocarpum*, which contain a 1, 4-dibenzodioxin moiety phenyl-substituted at positions 3 and 7 and belong to eckol series [25,46]. According to MS data, numbers 28 to 42 fall within eckol classes, and it was the first time eckol-type phlorotannins were detected in the *Sargassum* species. Among them, the molecular weights of numbers 28 to 39 all had two protons missing, when compared to corresponding fuhalol-type phlorotannins, which indicated the presence of one dibenzodioxin structural element in their structures. Besides, these compounds contained at least one hydroxyl group, so we categorized them as hydroxycarmalol series. Numbers 28 to 34 were predicted as phlorethohydroxycarmalols as repeoted by Glombitza et al. [46]. Moreover, numbers 35 to 39 were named as fuhalolhydroxycarmalols in our present study for the first time, because they had the same numbers of additional hydroxyl groups as corresponding fuhalols. It was essential to point out that the phlorotannins with molecular weights of numbers 28, 29, and 32 to 39 had never been reported in any previous studies and was detected for the first time in our present study, which still needed further separation and identification to elucidate the exact structures.

**Figure 4:**
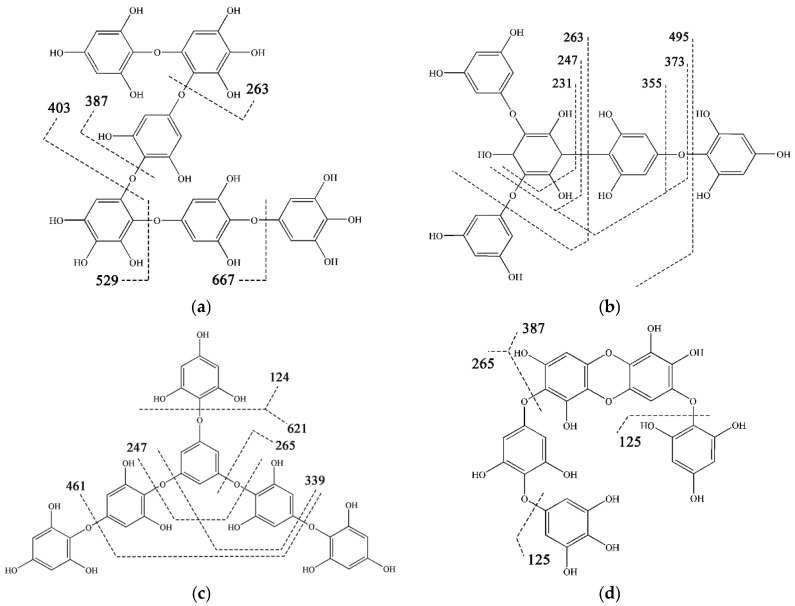
Structure of (**a**) molecular weight 794 (No. 5); (**b**) molecular weight 622 (No. 22); (**c**) molecular weight 746 (No. 23); and (**d**) molecular weight 652 (No. 36) tentatively identified in *S. fusiforme* extract and proposed fragmentation pathway.

In any case, the fragmentation pattern observed for the different molecular weights was very important for their identification, as they followed a typical fragmentation showing different losses of phloroglucinol units and hydroxyl groups, which helped to achieve a tentative identification [4]. In this study, the structures of phlorotannins of different molecular weights were tentatively identified using mass spectrometry. As shown in Figure 4, the examples of four classes of phlorotannins including fuhalol, phlorethol, fucophlorothol and eckol were illustrated. The first type of fuhalol-type phlorotannins was exemplified by the hexamer (No. 5, *m*/*z* 793, Table 1), the product ions of *m*/*z* 667, 529, 403, 387, and 263 might be due to the loss of phloroglucinol (126 amu), bifuhalol (266 amu), trifuhalol (390 amu), hydroxytrifuhalol (406 amu), and successive loss of one molecule of phloroglucinol, respectively. One of the proposed chemical structures of this hexamer was shown in Figure 4a, corresponding to hexafuhalol B as reported by Glombitza et al. [51]. The second type of fucophlorethol-type phlorotannins were exemplified by the pentamer (No. 22, *m*/*z* 621, Table 1), and its product ion peaks observed at *m*/*z* 495, 373, 355, 247, and 231 led to possible structures of fucotriphlorethol isomers (Figure 4b shows one of the proposed structures), which is probably due to the loss of one molecule of phloroglucinol (126 amu), two molecules of phloroglucinol (249 amu), two molecules of phloroglucinol and one water (266 amu), three molecules of phloroglucinol (374 amu), and successive loss of a molecule of water, respectively. The third type of phlorethol-type phlorotannins were exemplified also by the hexamer (No. 23, *m*/*z* 745, Table 1), the product ions of *m*/*z* 621, 461, and 339 might be due to the loss of phloroglucinol (126 amu), two molecules of phloroglucinol (250 amu), and three molecules of phloroglucinol and two molecules of water (406 amu), respectively. The proposed chemical structure of this hexamer shown in Figure 4c was corresponding to Hexaphlorethol-A as reported by Glombitza et al. [52]. Finally, the fourth type of eckol-type phlorotannins were exemplified by the pentamer (No. 36, *m*/*z* 652, Table 1), the product ion peaks observed at *m*/*z* 632, 387, and 265 led to possible structures of trifuhalolhydroxycarmalol isomers (one of the proposed structures were shown in Figure 4d), which is probably due to the loss of water (18 amu), bifuhalol (265 amu), and phlorethohydroxycarmalol (387 amu).

It was noteworthy that there were no previous studies describing in such detail of the phlorotannin composition in *S. fusiforme* samples or in any other algal sample, which contained a similar variability on fuhalols, deshydroxyfuhalols, phlorethols, fucophlorethols, eckols, and carmalol derivatives composition. In agreement with the previous reports, the dominant categories of phlorotannins in *Sargassum* species, including *S. muticum* [4,53,54] and *S. spinuligerum* [41,42,43,45,51,55], were fuhalol series, meanwhile some phlorethols and fucophlorethols were also elucidated. Furthermore, it is the first time that eckols and carmalol derivatives were detected in the *Sargassum* species. Some phlorotannins with molecular weights that were newly found in our study still need further separation and identification.

Our study provides evidence of antioxidant activities of *S. fusiforme* extracts/fractions based on in vitro tests. At present, there is little clarity as to the relationship between phlorotannin structure (degree of polymerization, type of bond, branching) and bioactivity. Some studies found that the antioxidant activity was correlated mainly with their phenolic contents, rather than the chemical structure or degree of polymerization of phlorotannins [4]. There were also some studies reported that the antioxidant activity is closely related to the number of free hydroxyl groups [56]. These two findings are not contradictory to each other, because most phlorotannins, although belonging to different categories, have similar structures, thus the number of hydroxyl groups mainly depends on the phlorotannin content rather than structure. This was in accordance with the results in our present study. Furthermore, the study of Fan et al. [57] indicated that hydroxyls in phlorotannins are hydrogen donors and vicinal-trihydroxyl is a more active hydrogen donor than meta-trihydroxyl. So, we can attribute the highly antioxidant activity of ethyl acetate fraction to its high phlorotannin content and high fuhalol-type phlorotannins composition which contain more vicinal-trihydroxyl elements.

## 3. Materials and Methods

### 3.1. Samples and Chemicals

The *S. fusiforme* sample was collected in 13 May 2014 in Dongtou (27°84′ N, 121°12′ E). The sample was washed with distilled water, then lyophilized. The dried seaweed was ground into powder and immediately stored at −20 °C until chemical analysis.

All solvents used were of HPLC grade. Phloroglucinol, 2,2-diphenyl-1-picrylhydrazyl (DPPH), 2,4,6-tris(2-pyridyl)-s-triazine (TPTZ), and 6-hydroxy-2,5,7,8-tetramethylchroman-2-carboxylic acid (Trolox) were purchased from Sigma-Aldrich (St. Louis, MO, USA). Folin-Ciocalteu’s phenol reagent and a tea polyphenol product with a purity of 90% were obtained from Solarbio (Beijing, China). All other reagents used in this study were of analytical grade.

### 3.2. Determination of Total Phlorotannin Content (TPC)

The TPC was determined using the Folin-Ciocalteu method described by Wang et al. [1] with a minor modification. 0.2 mL of sample solution was mixed with 1.3 mL of distilled water and 0.5 mL of Folin-Ciocalteu reagent. Then 1 mL of sodium carbonate (7.5% in distilled water) was added. The samples were incubated for 1 h at room temperature in the dark. The absorbance was measured at 770 nm with a UV-Vis spectrophotometer (UV-2102PC, Unico, Franksville, WI, USA). The phlorotannin content was calculated by using phloroglucinol (Sigma, St. Louis, MO, USA) as a standard (0–100 μg·mL^−1^), and the results were expressed as milligrams of phloroglucinol equivalents per gram of sample powder (mg PGE·g^−1^) or per 100 mg extracts (mg PGE/100 mg).

### 3.3. Determination of Extraction Parameters

#### 3.3.1. Initial Extraction Method

The extraction method of phlorotannins were performed as follows: 0.4 g of sample powder was dispersed in 50% ethanol solution at a solid/liquid ratio of 1:25 and incubated in a platform shaker at 50 °C for 1 h with a shaking speed of 125 rpm. The extract was filtered through a filter paper and the algal residue was washed with distilled water three times. Then the filtrates were combined and prepared for TPC determination. Each extraction was conducted in triplicate.

#### 3.3.2. Single Factor Experimental Design

The effects of different extraction variables, namely extraction solution (10, 30, 50, 70, and 90% ethanol solution), solid/liquid ratio (1:5, 1:10, 1:15, 1:20, and 1:25), extraction temperature (15, 25, 35, 45, and 55 °C), and extraction time (10, 20, 30, 60, and 90 min) on the TPC were studied by designing single factor experiments. In each experiment, the value of estimated factor was changed while those of other factors were performed using the conditions described in the Initial Extraction Method (Section 3.3.1). The best extraction parameters were chosen based on the highest TPC.

### 3.4. Preparation of Seaweed Extracts and Fractions

The solvent extracts were prepared according to the results from the Section 3.3. Briefly, 40 g of dried algal powder was dispersed in 200 mL of 30% ethanol solution, and incubated in a platform shaker for 30 min at room temperature with a speed of 125 rpm. The mixture was centrifuged at 2000 g for 15 min at 4 °C and the supernatant was collected. After extraction, the filtrate was collected and the solvent was removed by rotary evaporation. The residue was stored at −20 °C before use.

The crude 30% ethanol extract was subjected to solvent fractionation. The extraction method was the same as the procedure described above. The extract was concentrated in vacuum to about 100 mL, and the residue was partitioned three times with the same volume of ethyl acetate then 1-butanol, successively. After the solvent was removed and freeze-dried, three fractions—i.e., the ethyl acetate fraction, 1-butanol fractions, and an aqueous residue—were obtained.

### 3.5. Antioxidative Activity Assay

#### 3.5.1. DPPH Radical Scavenging Activity (DRSA)

The DSRA was determined according to Tierney et al. [5] with slight modifications. Prior to analysis, a working DPPH solution (0.10 mg·mL^−1^) and appropriate serial of the sample solutions were prepared using ethanol. In all experiments, Trolox, a synthetic antioxidant analogous to vitamin E, was used as the standard. The standard or sample (100 μL) and DPPH working solution (100 μL) were added to a 96-well plate. The plate was then left in the dark for 30 min at room temperature. The absorbance was measured at 515 nm on an automated Power Wave XS2 microplate spectrophotometer (Bio Tek, Winooski, VT, USA). The decrease in absorbance of the sample extract was calculated by comparison to a control. The ability to scavenge DPPH radicals was calculated using the following equation:

(1) $$\mathrm{Scavenging}\;\mathrm{effect}\;\left(\%\right)=\left[1-\left(A_{sample}-A_{sample\;blank}\right)/(A_{control}-A_{control\;blank})\right]\times 100$$

where *A_sample_* is the absorbance of the test sample (100 μL DPPH and 100 μL sample), *A_sample blank_* is the absorbance of the sample only (100 μL sample and 100 μL ethanol), *A_control_* is the absorbance of the control (100 μL DPPH and 100 μL ethanol), and *A_control blank_* is the absorbance of the ethanol only (200 μL ethanol). The IC50 value was calculated as the concentration of sample or standard antioxidant (μg·mL^−1^) requiring scavenging 50% of the DPPH in the reaction mixture.

#### 3.5.2. Ferric Reducing Antioxidant Power (FRAP)

FRAP assay was modified from that reported by Tierney et al. [5]. The oxidant in the FRAP assay consisted of a reagent mixture that was prepared by mixing acetate buffer (pH 3.6), ferric chloride solution (20 mM), and TPTZ solution (10 mM TPTZ in 40 mM HCl) in the ratio of 10:1:1. A trolox stock solution (2 mM) was prepared and diluted with ethanol to give concentrations ranging from 0.1 to 0.5 mM. Prior to analysis, the FRAP reagent was heated and protected from light, until it had reached a temperature of 37 °C. 180 μL of freshly prepared FRAP reagent at 37 °C was pipetted into a 96-well plate with either a 20 μL test sample or standard (or ethanol for the blank). The absorbance was measured at 593 nm after 50 min on an automated Power Wave XS2 microplate spectrophotometer (Bio Tek, Winooski, VT, USA). The trolox standard curve was used to calculate the antioxidant activity of the samples which was expressed as milligram trolox equivalents (TE) per milligram of sample (mg TE·mg^−1^).

### 3.6. UHPLC-QQQ-MS of Phlorotannin-Enriched Fractions

Chromatographic analyses were performed on an Agilent 1290 Infinity II UHPLC system (Agilent Technologies, Santa Clara, CA, USA) equipped with a multisampler (G7167B), an autosampler/injector, a high-speed pump (G7120A), a multicolumn thermostat (G7116B), a column compartment, and a diode array detector (G7117B) equipped with a 10-mm flow cell. The temperatures of the column oven and the autosampler were maintained at 30 °C and 4 °C, respectively. The autosampler is equipped with a black door, avoiding samples to be exposed to light. A Poroshell 120 EC-C18 column (2.1 mm × 50 mm, 2.7 μm, Agilent Technologies, Palo Alto, CA, USA) was used for separation. Mobile phases consisted of two solvents: aqueous solution (A) and acetonitrile (B). UV-vis spectra were scanned from 220 to 600 nm with a recording wavelength of 280 nm. The flow rate was 0.4 mL·min^−1^. The linear gradient was as follows: 5% B from 0 to 4 min, 10% B from 5 to 10 min, 10%–20% B from 10 to 15 min, 20% B from 16 to 20 min, 25% B from 21 to 25 min, 30% B from 26 to 30 min, 35% B from 31 to 35 min, 40% B from 36 to 40 min, 45% B from 41 to 52 min, and 45%–75% B from 52 to 55 min.

The UHPLC system was coupled to an Agilent 6460 triple quadrupole tandem mass spectrometry (Agilent Technologies, Santa Clara, CA, USA), controlled by MassHunter workstation software Version B.08.00. Ionization was performed using a conventional ESI source in positive and negative ionization modes. Other experimental conditions for the mass spectrometer were as follows: nebulizer, 15 psi; drying gas (N^2^) flow rate, 11.0 L·min^−1^; drying gas temperature, 350 °C; scan range, from 100 to 3000 *m*/*z*. In addition, MS/MS was used to capture and fragment the target ion we selected in the product ion scan.

### 3.7. Statistical Analysis

Each extraction and fractionation experiment was replicated three times, and analyses were performed three times. Measurement values were presented as means ± standard deviation. One-way analysis of variance (ANOVA), followed by the Tukey test, was carried out to test for significant differences in phenolic content and antioxidant activity using the statistical program IBM SPSS Statistics V19.0. A probability value of *p* < 0.05 was considered statistically significant.

## 4. Conclusions

In this study, an industrially feasible extraction and enrichment method of phlorotannins from *S. fusiforme* was established. The ethyl acetate fraction showed excellent antioxidant property and could be employed in the pharmaceutical industry as potent marine antioxidants. The UHPLC-QQQ-MS analysis revealed that phlorotannins in ethyl acetate fraction contained 42 molecular weights of compounds belonging to four classes of phlorotannins—including fuhalols, fucophlorethols, phlorethols and eckols—in which the fuhalol-type phlorotannins were the dominant compounds, and eckol-type phlorotannins, including some new carmalol derivatives, were detected and tentatively identified in *Sargassum* species for the first time. Further purification and structure elucidation of phlorotannin compounds are needed for a deeper understanding of the mechanisms behind the high antioxidant effects observed herein.

## Figures and Tables

**Table 1:** Mass spectrometric data of phlorotannins in the ethyl acetate fraction of *S. fusiforme* determined by UHPLC-QQQ-MS.

Categories	No.	DP ^1^	ESI	Measured Mass (*m*/*z*)	MS/MS Fragments Detected
**Fuhalols**					
	1	2-(1)	(−)	265	111, 123, 125, 139, 141, 247
	2	3-(1)	(−)	389	123, 125, 139, 245, 263, 265, 353
	3	4-(2)	(−)	529	123, 139, 245, 263, 389, 403
	4	5-(2)	(−)	653	139, 245, 263, 387, 389, 513, 527
	5	6-(3)	(−)	793	139, 263, 387, 389, 403, 529, 667
	6	7-(3)	(−)	917	373, 387, 527, 653, 785
	7	8-(4)	(−)	1057	262, 527, 543, 793, 917
	8	9-(4)	(−)	1181	
	9	10-(5)	(−)	1321	
	10	11-(5)	(−)	1445	
	11	12-(6)	(−)	1585	
	12	4-(1)	(−)	513	246, 265, 373, 389
	13	5-(1)	(−)	637	123, 247, 265, 373, 388, 511
	14	6-(1)	(−)	761	140, 261, 263, 355, 387, 389, 512, 621, 635
	15	6-(2)	(−)	777	124, 245, 387, 389, 402, 513, 636
	16	7-(1)	(+)	887	
	17	7-(2)	(−)	901	262, 387, 511, 513, 527, 635, 637
	18	8-(3)	(−)	1041	245, 263, 387, 389, 511, 513, 527, 621, 653, 777, 901
	19	9-(3)	(−)	1165	
	20	10-(4)	(−)	1305	
**Phlorethols/Fucols/Fucophlorethols**					
	21	2	(+)	251	93, 109, 123, 139, 233
	22	5	(−)	621	139, 231, 247, 263, 355, 373, 495
	23	6	(−)	745	124, 247, 265, 339, 461, 621, 727
	24	8	(−)	993	
	25	9	(−)	1117	
	26	10	(−)	1241	
	27	11	(−)	1365	
**Eckols**					
Carmalol Derivatives	28	2-(1)	(−)	263	111, 219, 245
	29	3-(1)	(−)	387	123, 245, 262, 329
	30	4-(1)	(−)	511	
	31	5-(1)	(−)	635	
	32	6-(1)	(−)	759	
	33	7-(1)	(−)	883	
	34	8-(1)	(−)	1007	
	35	4-(2)	(−)	527	123, 233, 261, 263, 139, 403
	36	5-(2)	(−)	651	125, 265, 244, 387, 632
	37	6-(3)	(−)	791	261, 356, 385, 747
	38	7-(3)	(−)	915	263, 387, 652, 681, 791
	39	8-(4)	(−)	1055	
Eckol	40	3	(−)	371	121, 140, 229, 246, 317, 335
Dieckol	41	6	(−)	741	389, 600
Dioxinodehydroeckol	42	3	(+)	371	121, 236, 248, 267, 335, 353

^1^ DP was the abbreviation of the degree of polymerization. In the expression of a-(b), a means the degree of polymerization, and b means the number of additional hydroxyl groups.
